# Bayesian regression versus machine learning for rapid age estimation of archaeological features identified with lidar at Angkor

**DOI:** 10.1038/s41598-023-44875-0

**Published:** 2023-10-20

**Authors:** W. Christopher Carleton, Sarah Klassen, Jonathan Niles-Weed, Damian Evans, Patrick Roberts, Huw S. Groucutt

**Affiliations:** 1 Extreme Events Research Group, Max Planck Institutes of/for, Geoanthropology, Chemcial Ecology, and Biogeochemistry, Jena, Germany; 2 Department of Anthropology, University of Toronto, Toronto, Canada; 3 Courant Institute of Mathematical Sciences and Center for Data Science, New York University, New York, USA; 4 École française d’Extrême-Orient, Paris, France; 5 isoTROPIC Research Group, Max Planck Institute of Geoanthropology, Jena, Germany; 6 Department of Archaeology, Max Planck Institute of Geoanthropology, Jena, Germany; 7 Department of Classics and Archaeology, University of Malta, Msida, Malta; 8 Institute of Prehistoric Archaeology, University of Cologne, Cologne, Germany

**Keywords:** Environmental sciences, Environmental social sciences, Mathematics and computing

## Abstract

Lidar (light-detection and ranging) has revolutionized archaeology. We are now able to produce high-resolution maps of archaeological surface features over vast areas, allowing us to see ancient land-use and anthropogenic landscape modification at previously un-imagined scales. In the tropics, this has enabled documentation of previously archaeologically unrecorded cities in various tropical regions, igniting scientific and popular interest in ancient tropical urbanism. An emerging challenge, however, is to add temporal depth to this torrent of new spatial data because traditional archaeological investigations are time consuming and inherently destructive. So far, we are aware of only one attempt to apply statistics and machine learning to remotely-sensed data in order to add time-depth to spatial data. Using temples at the well-known massive urban complex of Angkor in Cambodia as a case study, a predictive model was developed combining standard regression with novel machine learning methods to estimate temple foundation dates for undated Angkorian temples identified with remote sensing, including lidar. The model’s predictions were used to produce an historical population curve for Angkor and study urban expansion at this important ancient tropical urban centre. The approach, however, has certain limitations. Importantly, its handling of uncertainties leaves room for improvement, and like many machine learning approaches it is opaque regarding which predictor variables are most relevant. Here we describe a new study in which we investigated an alternative Bayesian regression approach applied to the same case study. We compare the two models in terms of their inner workings, results, and interpretive utility. We also use an updated database of Angkorian temples as the training dataset, allowing us to produce the most current estimate for temple foundations and historic spatiotemporal urban growth patterns at Angkor. Our results demonstrate that, in principle, predictive statistical and machine learning methods could be used to rapidly add chronological information to large lidar datasets and a Bayesian paradigm makes it possible to incorporate important uncertainties—especially chronological—into modelled temporal estimates.

## Introduction

Lidar (light-detection and ranging) has become widely appreciated as a revolutionary new tool for archaeological discovery and heritage management^1^. It is a sophisticated laser scanning technology that can be used to produce 3D models of the Earth’s surface even through dense forest canopies. In 2011, a lidar surface map of an historically important Classic Maya centre in Belize called Caracol was published^2^. The lidar scanning covered 200 km^2^ and revealed as many as eleven new causeways and thousands of previously unrecorded residential buildings and agricultural terraces. It provided a new thorough mapping of this complex, vast Maya megalopolis beneath the tropical canopy that was too difficult to make out and comprehend at ground-level. This was the latest in a long line of archaeological studies in Mesoamerica spanning decades that undermine long-standing notions that tropical forests, broadly speaking, were too challenging, too hostile, too nutrient- and protein-poor to support large, complex societies [e.g. Ref.^3–5^] (and see Ref.^6^). In 2013 a lidar survey was published revealing the previously unmapped extent of Angkor in Cambodia, the centre of power of the Khmer Empire from 800 to 1400 CE^7^. That survey identified thousands of previously unmapped features including temples, reservoirs, and causeways indicating that the ancient city covered at least 1000 km$^{2}$ with no obvious drop-off in structure density, confirming Angkor’s position as the world’s largest pre-industrial urban complex by area anywhere in the world, let alone a tropical setting^8^. Even more recently, lidar scanning has also now revealed ancient cities in the Amazon basin^9^. There will undoubtedly be more to come since the Amazon basin covers some 6.3 million km^2^, roughly a third of the South American continent. At the same time, lidar studies continue in the Classic Maya region [e.g. Ref.^10^] and will certainly be extended to other tropical areas.

While scientifically transformative, lidar and other remote-sensing technologies are only capable of producing a spatial palimpsest of ancient societies, effectively the sum-total footprint of their land-use and landscape modification. As more surveys are conducted and more ancient features are brought to light, the next challenge will be to find a way to make good use of the flood of spatial data. In order to, for example, answer questions about the interaction between ancient people and their environments through time—e.g. responses to climate change, population trajectories, urban development and sustainability—we need to add a temporal dimension to the lidar data and other forms of archaeological survey. While ground-survey and excavation will continue to be key, it is time-consuming and practically impossible across the entire scale of mapped urban areas. In 2018, Klassen et al.^11^ published the first attempt to address this challenge with novel machine learning tools. They applied those tools to add temporality to temples identified by pedestrian and remote sensing survey at Angkor.

**Figure 1:**
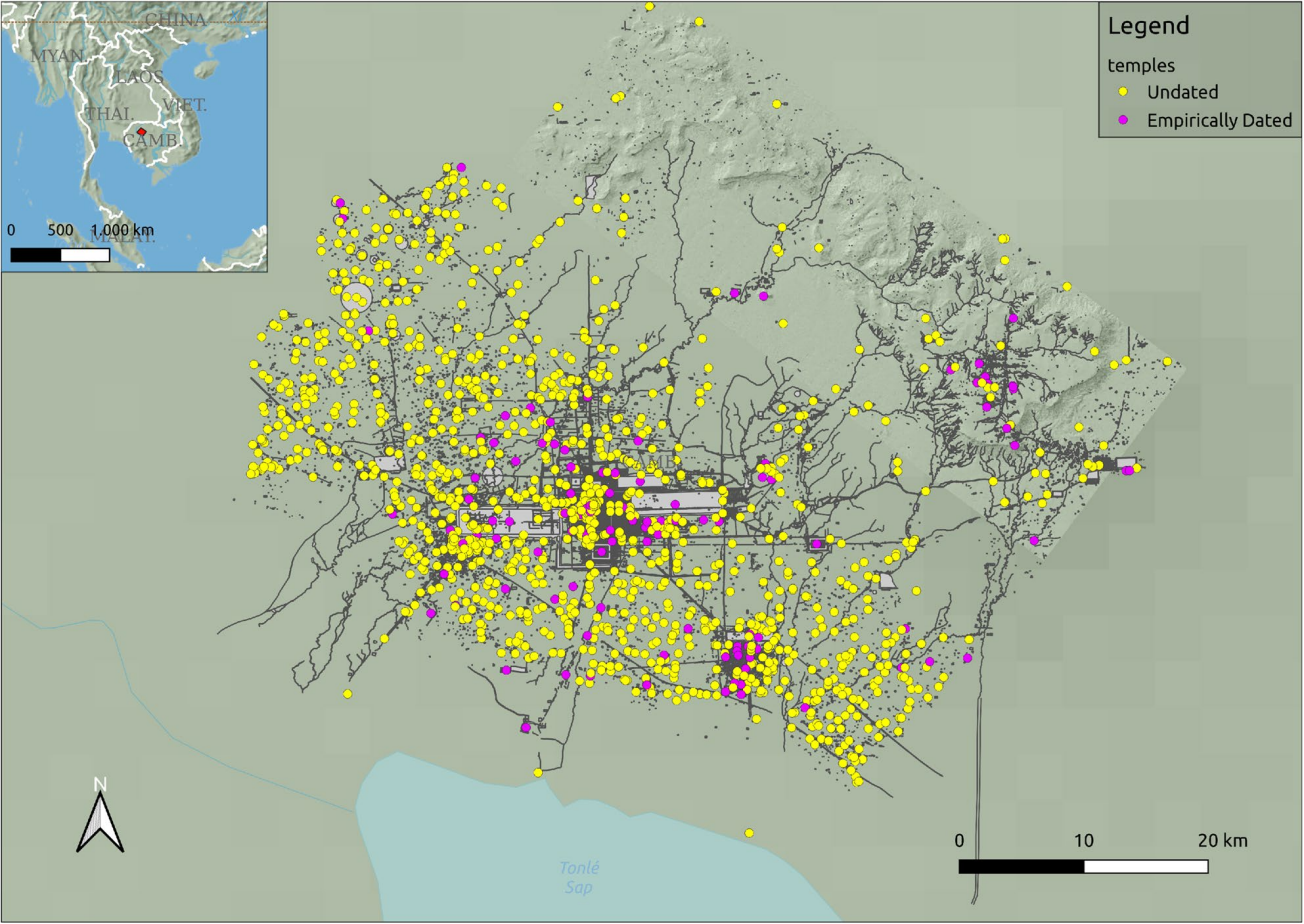
Map showing location of Angkor and the distribution of temple locations for data used in this study. This map was created with QGIS 3.28.10 (https://www.qgis.org). Point data for temple locations and vector data for Angkor’s infrastructure (roads, reservoirs, etc) were provided by three of the authors (SK, JW, and DE) and first reported in^11^; inset map hillshade and vector data are in the public domain and free for use from Natural Earth (https://www.naturalearthdata.com/)^25^; main map hillshade was derived from lidar data^7^ and created in QGIS.

Temples were a central political and economic organizing force that helped shape the history of Angkor^12,13^. The capital of the Khmer Kingdom for most nearly 600 years^13,14^, Angkor is situated in the northwest of present-day Cambodia on the north side of the great lake, Tonlé Sap (see Fig. 1). The city may have been home to as many as 900,000 inhabitants at the height of its influence and prosperity^15^. Unlike most other cities at that time, Angkor’s population was spread out over more than 1000 km$^{2}$ encompassing a complex hydrological management system comprising hundreds of kilometres of canals and channels^7,16^ and a web of roads and embankments connecting ancient Angkorians in a vast urban network^14^. The city included a patchwork of administrative, religious, domestic, and agricultural spaces, leading scholars to refer to it as ”agro-urban” or ”low-density agrarian urbanism” [e.g. Ref.^17,18^]. Over 1400 temples have been identified in Angkor’s metropolitan area through a combination of satellite imagery and ground and the aforementioned lidar surveys^7,19,20^ (see Fig. 1). Social, political, and economic activity in this massive agro-urban complex was oriented around these temples, which were vital to developing and maintaining the city’s hydraulic infrastructure, economy, political power structures, and population^12,13,16,21–24^. Thus, understanding the growth and development of the temple system is vital for investigating the evolution of Angkor as a whole.

Using a training sample of temples at Angkor with known founding dates, Klassen et al.^11^ attempted to predict the founding dates of the hundreds of un-dated temples identified over decades of pedestrian surveys and in the 2012 lidar survey. Their premise was that observable traits might be useful for predicting temple founding dates, meaning that they could rapidly add temporal information (albeit estimates) to the recently acquired lidar data, especially newly identified habitation features associated with the temples. This notion, that urban developmental trajectory could be reconstructed by leveraging observable temporal patterns, has antecedents in archaeological, historical, and urban studies research—e.g. Lilley’s^26^ work on using street/property grid orientations to identify stages of urban development in English medieval cities. Klassen et al.’s dataset comprised 1431 temples, 105 of which had dates derived from inscriptional or art-historical analyses. They recorded several variables, including temple shape, the presence of a moat, the presence of a primary reservoir, construction materials, major axis orientation (azimuth), and total area of the temple and associated grounds. Some of these variables were collected over decades of pedestrian survey and others, like temple morphology and major axis orientation, were derived entirely from remotely sensed data. Klassen et al.^11^ then evaluated a handful of several quantitative approaches that might be used to predict missing foundation dates, including k-Means Clustering, Discriminant Function Analysis, Generalized Linear Modelling (GLM), and a Graph-Based Semi-Supervised Learning (GSSL) method called ”label propagation”. Using leave-one-out cross-validation, they found that the best overall predictions were obtained by combining the results of GLM with label propagation.

While their assessment indicated that the premise is sound—i.e. we can predict temple dates quantitatively to rapidly add temporal information to lidar-derived and other remotely sensed data—the approach has two limitations. Chiefly, the predictions are made without incorporating or propagating uncertainty. The hybrid GLM-GSSL approach produces a single best foundation date estimate for each temple. It cannot account for variability in the relationship between the predictor variables and the dates, the uncertainty in the underlying observations, or the uncertainty in predictor variable imputations. This last source is particularly important because only 73 of the 1431 available temples had values for all predictor variables. Ideally, these sources of uncertainty would be included in the prediction and represented by a distribution of probable dates for each temple rather than a single estimate so that the uncertainty can be propagated into further analyses. The other limitation is that label propagation, like many semi- and unsupervised machine learning methods, can be opaque regarding which patterns were used to make predictions. In the GSSL paradigm, the relevant patterns are related to ”structure” in the graph used to represent the data^27^. This ”structure” can refer to anything from trends, to clustering, to isolated neighbourhoods (where a group of observations are more similar to each other than others), and more. The similarity is defined by a metric that combines information across all variables in the model with no way to distinguish their individual contributions. Consequently, it can be impossible to determine which ”structure” was used to improve predictive accuracy or which variables were the most relevant for determining that structure. When the aim is purely predictive, the opacity is not a problem. But, if there are alternative approaches that can predict and explain (in the statistical sense) variation, we can get even more information out of our data.

Considering these limitations, we explored an additional approach involving Bayesian regression that could also be applied broadly to lidar-derived datasets. Bayesian analyses are based on probability theory and usually concern full posterior distributions for model parameters^28,29^. The paradigm is, therefore, fundamentally about uncertainty quantification, expression, and propagation. Additionally, most modern Bayesian analyses involve Monte-Carlo simulation, which is an iterative procedure for exploring model likelihoods that rely on randomness^30^. As part of the process, parameter values are sampled repeatedly. This means that one can treat all variables as parameters to be sampled and then define sampling distributions for variables missing from some observations. Consequently, uncertainty about the true values of those missing variables can be encoded and then propagated into the estimated posterior distributions. For present purposes, this means we could include uncertainty about missing temple variables, and that uncertainty will be reflected in the model’s estimate for the relevant temples’ foundation dates. Lastly, the Bayesian model we explored is a simple regression model, which means we can assess the relative importance of predictor variables using a standard statistical toolkit. We can thereby reveal potentially important and interesting relationships between the historical trajectory of temple foundation events and the variables used to predict the relevant dates. Here we present the results of a study in which we compare the hybrid GLM-GSSL model’s predictions with those of the Bayesian regression model. Importantly, we applied both models to a new and updated database of Angkorian temples and, as a result, produced the most up-to-date trajectories for temple foundation dates published so far. We also fully explore and compare the two approaches so that they can be further developed and applied to other archaeological case studies involving lidar and ground survey data.

## Results

**Figure 2:**
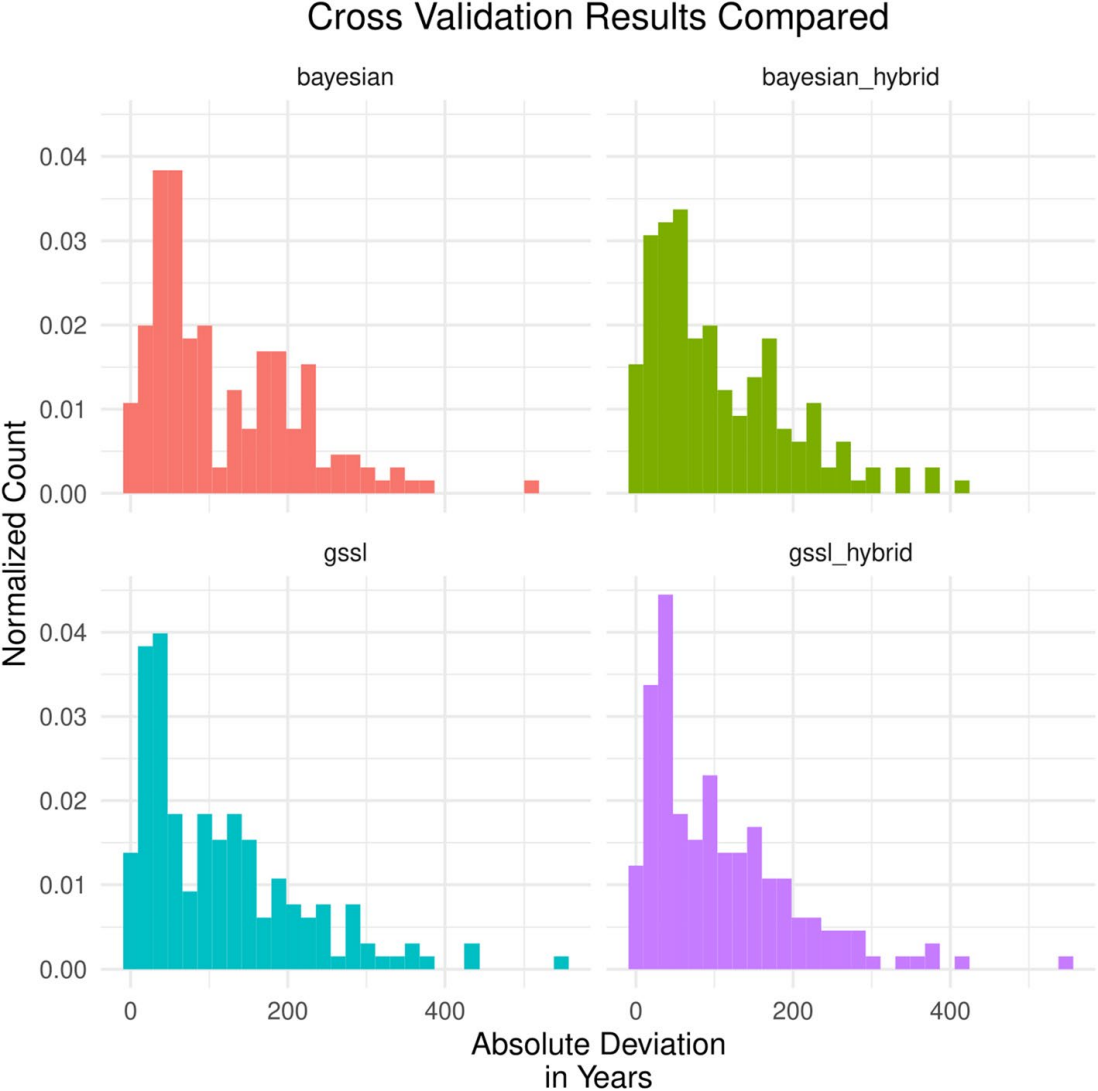
Histograms of absolute deviations. The top row displays the deviation for the Bayesian model and the bottom row displays the deviations for the GSSL model. For the sake of comparison, we included un-hybridized and hybridized results for both models (see "Methods").

The cross-validation analyses revealed that the two approaches have similar predictive performance in terms of absolute deviations. The GSSL model had a mean absolute deviation of 113 years, while the Bayesian model’s was 116 years, and the median deviations were 87 years and 82 years, respectively. The distributions were also similar overall (see Fig. 2)—note that we have also included a plot where we ”hybridized” the Bayesian model with the GLM results in order to make the results as comparable as possible.

The results of the Bayesian variable selection analysis were highly enlightening (see Table 1). Only two of the predictor variables significantly improved the overall likelihood of the model. One relevant predictor was the presence of ”brick” at the temple, which had an inclusion probability of one. This meant that the RJ-MCMC always included that variable throughout the simulation. The other relevant variable was the presence of ”*thma phom*” as a building material—*thma phom* is Khmer for ”mountain stone”, referring broadly to igneous rubble, like rhyolite. However, it had a lower inclusion probability at around 0.61, which suggests its influence is much less useful than the presence of ”brick” for determining temple foundation dates. No other predictor variables had inclusion probabilities higher than 0.25, with most less than 0.15.

**Table 1:** Variable selection results; RJ-MCMC inclusion probabilities.

Nimble parameter	Variable	Description	Inclusion prob.
Beta[1]	Azimuth	Azimuth	0
Beta[2]	Area	Area	0
Beta[3]	Trait 1	Principle reservoir	0.03
Beta[4]	Trait 2	Moat	0.13
Beta[5]	Trait 3	Sandstone	0.08
Beta[6]	Trait 4	Pink sandstone	0.15
Beta[7]	Trait 5	Laterite	0.1
Beta[8]	Trait 6	Brick	1
Beta[9]	Trait 7	*Thma phnom*	0.61
Beta[10]	Trait 8	Other	0.25

Interesting patterns also appeared in the posteriors of the morphotype parameter of the Bayesian model. The posterior distributions indicate that some morphotypes are older than others and that there was change over time in the layouts of newly founded temples. On average, temples with the ”square” morphotype were the oldest, followed by ”causeway-4” types, ”horseshoe-east”, and then ”causeway-2” and ”blob” types. However, there was significant overlap between many of the types, and some of these differences in mean foundation date were very slight. There were also too few examples of ”circle” and ”horseshoe-north” types in the training dataset to be certain about their relative positions in the morphotype chronology.

**Figure 3:**
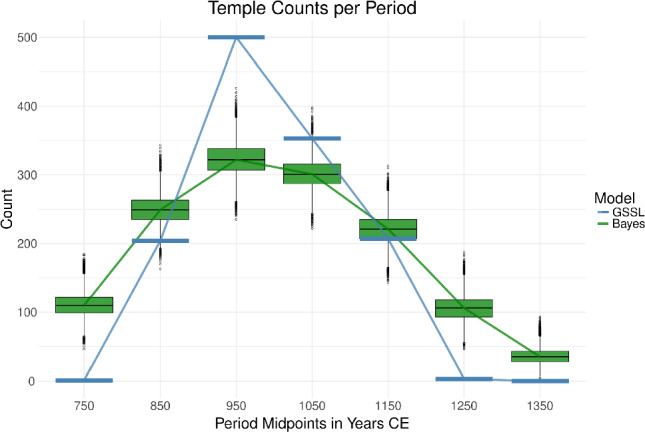
Estimated temple foundation events per century based on counts derived from each model. The Bayesian model includes chronological uncertainty, which results in count per period being a random variable with corresponding uncertainty represented by standard box and whisker plots.

The visualizations were also revealing. Plotting the counts of temple foundation events per period exposed important differences between the predictive approaches despite some overall similarity in the trajectories (see Fig. 3). Only the plot produced with the Bayesian model included information about temporal uncertainty propagated into the per-period counts. The box plots represented this uncertainty. In contrast, the GSSL model produced a single point estimate per temple and, so, only one trajectory for temple foundation events. The counts produced using the GSSL model would be considered highly improbable from the view of the Bayesian model in five of the seven periods considered. Moreover, the peak in foundation events is more sharply defined in the GSSL curve, and the rates of increase at the beginning of the series and decrease at the end are higher than those rates suggested by the Bayesian model. The Bayesian model also suggests that many more temples may have been founded in the first century of the sequence and the last two centuries than indicated by the GSSL model.

**Figure 4:**
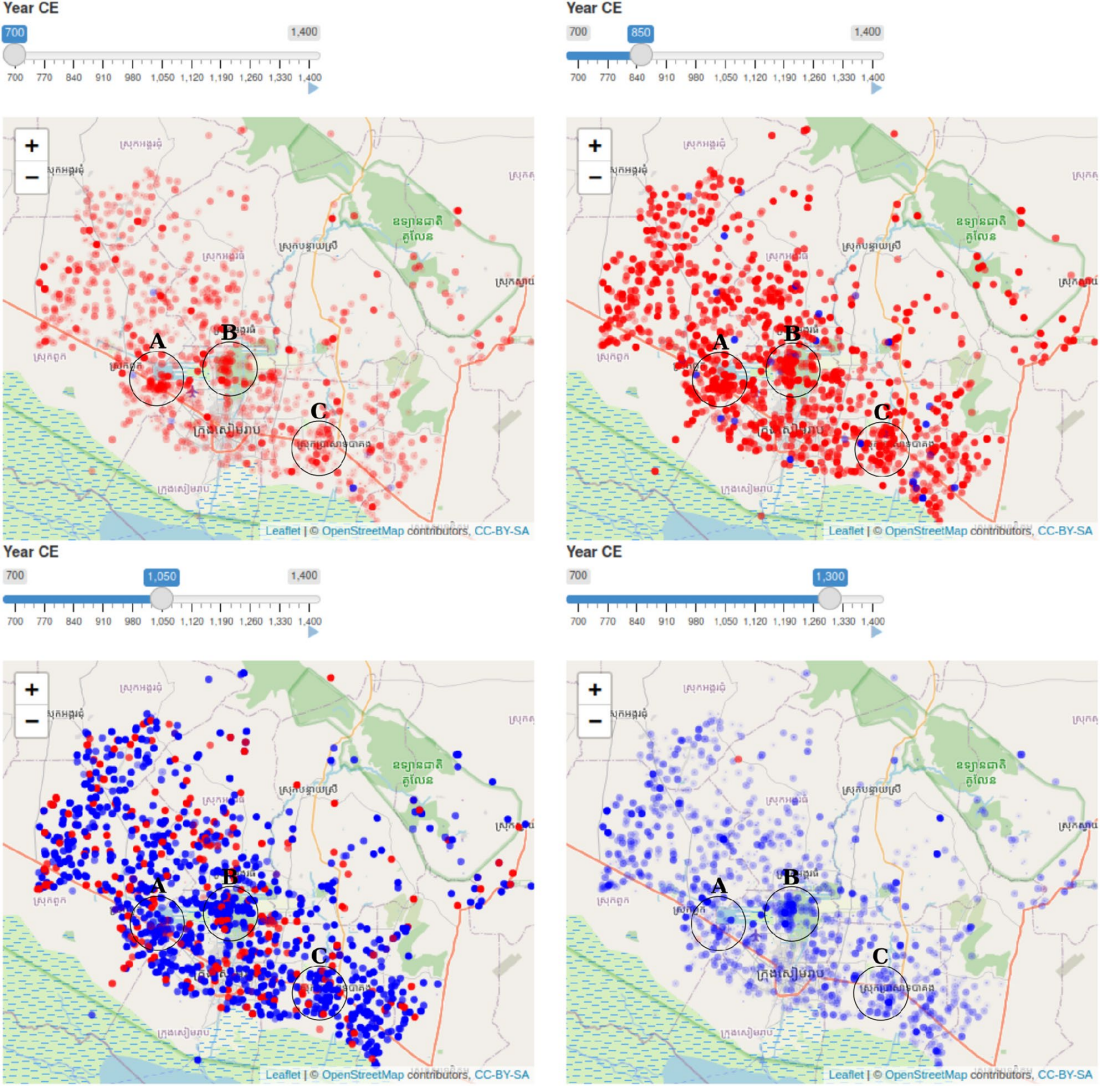
Temporal snapshots of temple foundation events. Temple locations are indicated by the blue and red circles. At the top of the app window is a slider widget that sets the date for the visualization. Temporal uncertainty about foundation date is indicated by symbol opacity—as the opacity increases, so does the probability that the corresponding temple was founded at the time set by the slider widget. The colour of a given circle indicates whether the mean estimated foundation date for that temple is earlier (blue) or later (red) than the date set by the slider. The three circles indicate areas mentioned in the text.

The spatial visualization revealed interesting spatial patterns in the temple foundation process (Fig. 4). At the beginning of the period, around 700 CE, there are three visually distinct clusters of foundation events with relatively high-probabilities with a scattering of other foundations at some distance from the core of Angkor. The initial high-density areas are labelled A, B, and C in Fig. 4 and they correspond to the locations of Bhavapura, Yaśodharapura, and Hariharālaya, respectively. All three were important early Angkorian cities^31^, and area B also came to be the location for the great walled city of Angkor Thom in the 12th century^13^. Within a few centuries, temple foundations begin to fill in the landscape until, half way through the period, most of the greater Angkor metropolitan area contains at least some high-probability foundation events with some obvious low-density regions interspersed throughout the overall area. Still, even at the peak of foundations, the highest density areas from the beginning continue to contain foundation events. Then, towards the end of the period, rather than seeing a continuous in-filling of relatively lower density areas, it is again a handful of already high-density spots that appear to be the epicenters of further foundation events, especially the area labelled B in the images corresponding to Angkor Thom, the last major Angkorian period capital of the Khmer Empire.

## Discussion

At first glance, the models appear to have had similar predictive utility. Both had mean absolute deviations of around 114 years and median deviations of around 84 years. This was a surprising finding, given the way that the GSSL approach handles missing data imputation.

Take, for example, the imputation of missing data in the case of the presence/absence of moats. Like the other binary traits, the trait ”moat” could be present or absent with missing values coded as ”na”. For the GSSL approach, this binary variable with missing values allowed was converted into effectively a trinary response: ”true”, ”false”, and ”na” where the last possibility was treated as a third discrete category rather than as an unknown. If two given temples had the value ”na” for the trait ”moat”, they were automatically considered more similar because the ”na” was treated as equal to ”na”. However, imagine that 50% of the temples really have moats. Then, the imputation will lead to a correct assessment of similarity only 50% of the time. Add another presence/absence variable, and the probability that the two imputations together would lead to a correct assessment of similarity declines to 25%. Add a third, and the probability declines to 12.5%, and so on. Increasing the number of predictors rapidly increases the chances that the imputation would be wrong with implications for the estimated similarity between two temples with missing data. More importantly, the imputation would be *confidently wrong* because the model does not include imputation uncertainty.

This confidence in wrong answers is almost bias by definition, raising an interesting question about why the two models appeared then to have similar predictive utility. The reason, we think, is that the predictor variables included in the analysis were generally not predictive of foundation date anyway. As the variable selection analysis revealed, only the presence of two building materials—brick and *thma phom*—were useful for predicting temple foundation dates in the training sample. Furthermore, only one or two of the temple morphotypes appear to have differed significantly from the rest. Consequently, the biased imputation for the GSSL model would have had little effect with respect to most of the variables examined. It is perhaps not surprising that reducing the impact of those biases with a Bayesian approach did not radically alter predictions, leading to similar mean absolute deviations between the two approaches.

That said, the mean absolute deviations are just one point of comparison, and looking at other similarities and differences is more revealing. Take, for instance, the modelled foundation trajectories. Both modelled trajectories show an increase in temple foundations from the beginning of the examined period (700 CE) followed by a crest and decline toward the end of that period (1400 CE). However, the initial level, rate of incline, height, and definition of the middle peak, and rate of decline in foundation events all differ between the models. The GSSL model implies a very rapid increase in temples in the study area from almost none in the 8th century to over 400 in the 10th century, followed by a rapid decline to nearly zero foundations in the 13th century. By contrast, the Bayesian model suggests a range of temple counts was possible in all centuries, but a much more gradual median trajectory throughout the observation period. It estimates that the process began with a median of over 100 temples founded in the 8th century. Then, the process inclined more gradually over the next two hundred years, rounding off at just over 300 temples per century in the 9th and 10th centuries, a much more level ”peak” in abundance. Finally, the Bayesian model predicts a gradual decline in foundation events with a median count of still around 100 foundations in the 13th century when the GSSL predicted there were nearly zero. In only the 12th century could the Bayesian model be fairly said to agree with the GSSL-hybrid estimate. In each other case, the GSSL-hybrid based counts would be considered extremely unlikely from the Bayesian model’s perspective. Consequently, correlations found between the GSSL-based temple foundation count trajectory (or extensions of it) and other data would also be deemed less likely than they appear from view of the Bayesian estimates. Such differences would become particularly important for high-resolution comparisons between temple foundation trajectories and climatic or historical datasets because the short-term patterns might matter more.

As noted, the Bayesian approach also has certain other scientific advantages. One is that the importance of predictive variables can be easily assessed. In doing so, we found that the presence of brick and *thma phom* have predictive utility. The model’s posteriors also tells us, though, about which way the age estimates move on average given the presence of each material—i.e. the marginal effect on temple foundation date for each variable. Brick, for instance, was used more frequently in early temple construction, leading on average to foundation estimates that were 150 years older for temples with brick holding other variables constant—a finding in line with earlier research indicating temporal patterns in construction material of temples at Angkor and in Southeast Asia more generally^32,33^. This also means, though, that square temples containing brick should be, on average, the oldest temples in the Angkor region, leading to a straightforward prediction for empirical verification and raising interesting questions about the trajectory of temple architecture concerning the combination of both form and material.

The other major advantage of the Bayesian approach is that the temple foundation dates are provided with full uncertainties in the form of posterior distributions. These distributions can then be used in further analyses, like population projections [e.g. Ref.^15^], or to quantitatively test hypotheses about the impact of climate changes [e.g. Ref.^34,35^] or historical events and process [e.g. Ref.^36,37^] on the historical trajectory of temple foundations at Angkor. Importantly, the uncertainty the estimated foundation-date distributions represent can be carried along and propagated into whatever estimates are produced by subsequent analyses. So, for instance, studies like Buckley et al.^35^ that explored the relationship between climate change and the historical trajectory of Angkor could be revisited and the comparison between climate data and historical urban trajectory could now include the chronological uncertainties indicated by the Bayesian model. Alternatively, the Bayesian model itself could be extended. It could be one component of a much larger Bayesian model that includes parameters derived from temple foundation event counts (like population projections or economic indicators) and uncertainties in other time series data of interest (like climate records).

**Figure 5:**
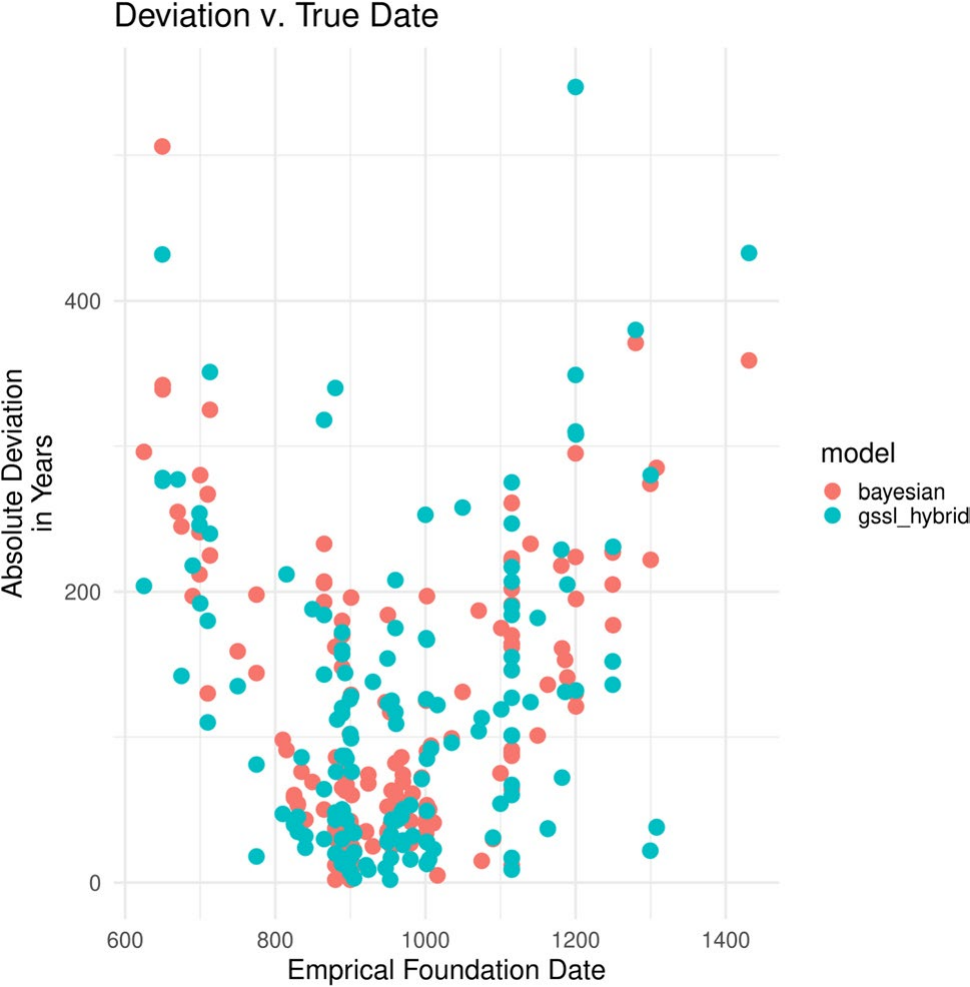
Prediction deviations (prediction error) plotted against known temple foundation date. Note that the Bayesian model actually predicts a distribution of ages for each temple and only the mean predictions are shown here for comparison with the GSSL-hybrid predictions.

It is important to point out, nonetheless, that both models suffer from some of the same limitations. Chiefly, biases in the training sample will propagate into the predictions of both models. The only data relating foundation dates to temple traits come from the dated temples. Consequently, strong biases in the sample of dated temples could lead to poor out-of-sample estimates regardless of the approach used. Such a bias can be seen in the predictions of both models we evaluated when comparing their cross-validation predictions to the known foundation dates of temples in the training dataset (see Fig. 5). In both cases, we can see that prediction error is higher for older and younger temples. This pattern probably occurred for two reasons: (1) there are simply fewer older and younger temples in the training data on which to base predictions for undated older and younger temples, respectively; and (2) the relationship between predictors (temple traits) and the foundation dates may have changed over time, meaning that the models have less predictive utility for temples of certain ages. The only solution is to gather a bigger training sample, particularly including temples founded during the earlier and later periods, and verify model predictions with chronometric methods like radiocarbon or optically-stimulated luminescence. So, while the predictive approaches we evaluated are essential—especially in the context of temple foundation dates where a complete ground survey of over 1400+ temples just is not feasible—they are only models. It is worth remembering George Box’s admonition that ”...all models are wrong, but some are useful...”^38^.

Our research has implications for the recent and likely ongoing explosion of lidar-based archaeological discoveries in the tropics. The lidar revolution in archaeology is drastically improving, and sometimes radically altering, our understanding of past tropical societies^1,39^. Lidar programs in Mesoamerica^39^, Cambodia^7^, and most recently the Bolivian Amazon^9^, have demonstrated conclusively that complex urban societies form in tropical environments, and that the global tropics have been home to some of the most extensive urban societies ever recorded^40^. Importantly, the lidar data has revealed unambiguous evidence for those societies having modified their environs extensively, adding to a growing body of evidence for a long-term, and widespread human modification of the global tropics^6^. A major challenge, and we think exciting opportunity, going forward will now be providing time-depth to complement the spatial extent of the lidar data. Of course, a key component of that work will be fieldwork and dating methodologies applied to specific regions of interest in order to build up training data necessary for chronological predictions. And, importantly, the specific feature traits and dating methods will have to be tailored to target regions in order to reflect context-specific information availability. Not all regions will have, for instance, epigraphically datable temples of varying shapes and construction materials and, so, other feature specific temporal patterning would be required. The approaches we explored can be used in any situation where potential chronologically predictive feature traits are available, which could include variables that can be gathered exclusively with remote sensing technology like morphology, orientation, geophysical context, volume, footprint size, or spatial associations with other features, all of which could be identified in lidar data either manually or perhaps with machine learning tools. Ground-survey acquired data may also be predictive where available and could hypothetically include surface artifact concentrations and/or assemblage information of the kind commonly gathered during ceramic pedestrian surveys for instance. Given the presence of temporal patterns and sufficient training data, the study by Klassen et al.^11^ and the one we present here clearly demonstrate that chronological predictive approaches could be used to economically provide age estimates for archaeological features and sites identified in lidar surveys. These techniques could allow us to rapidly develop a spatiotemporal understanding past human-environmental interaction and trace the spatiotemporal trajectories of tropical urbanism around the world.

Future research, in our view, should proceed in at least three directions. The first is further empirically testing model predictions about foundation dates. This will entail randomly sampling temples and deploying a combination of chronological methods to produce evidence-based foundation dates for comparison with corresponding model predictions. As this research goes on—likely in tandem with other field-based research at Angkor—the model(s) can be continuously evaluated and updated, as we have done here by including newly available training dataset. It would also be enlightening to examine any deviations between model predictions for specific temples to investigate the potential importance temples that may appear to be chronological outliers with respect to one of the models. Another direction for future research should be the continued search for, and evaluation of, alternative modelling approaches. There have been, for instance, developments toward Bayesian graph-based semi-supervised learning that could seamlessly handle missing data and account for uncertainty in the structure of the graph^27^. When the theoretical foundations of this emerging approach have been appropriately laid and stress tested (with simulation, for example), and appropriate software has been developed, it could be used to create new predictions for temple foundation dates that take the best of both approaches we evaluated. Machine learning, and particularly AI, approaches have enormous potential for helping to resolve archaeological research questions and expose patterns in pre-existing data that will lead to new questions, and we are hopeful that both approaches we considered will be superseded by even better ones in the future. And, lastly, chronological predictive approaches like the ones explored here should be deployed in other tropical environments with available archaeological lidar data, like Mesoamerica and the Amazon basin, in order to add time-depth to the emerging picture of ancient tropical urbanism around the globe.

## Methods

### Temple data

The original temple dataset from Klassen et al.^11^ included 1431 temples, of which 105 had founding dates. Since that paper was published, additional research has revealed founding dates for a further 58 temples bringing the total number to 163^15^. Some of these 163 temples had their consecration dates commemorated in ancient Khmer or Sanskrit inscribed directly into architectural elements or associated stone stelae^22^. Such inscriptions provide fairly direct chronological evidence for founding a new temple. Even though it is possible that the actual construction date and the religious consecration date may have been different, the discrepancy is unlikely to be very large in most cases. Other temples may only have inscriptions indicating the date of a particular transaction, or reference to another significant event. In these cases, the earliest date in evidence could be used to estimate the temple’s foundation date with the caveat that such a date is only a *terminus ante quem*. Still, other temples can be dated indirectly by comparing certain art-architectural elements with those of dated temples^41,42^. Consistent styles of relief sculpture on door lintels, for example, emerged at different times^41^. An undated temple could, therefore, be assigned a date based on similarities between its door lintel designs and those of a dated temple. The 163 dated temples formed the new/updated training dataset used in all of our analyses so that the results from the two models would be directly comparable.

The database used included 11 predictors, as in Klassen et al.^11^. The first was temple morphology, a categorical variable with 8 categories: ”square”, ”circle” , ”two-causeways”, ”four-causeways”, ”horseshoe (north)”, ”horseshoe (east)”, ”horseshoe (west)”, and ”blob”. The second predictor was azimuth, i.e. the degrees off north of the axis of primary access to the temple as determined in a GIS package with remotely sensed data—manually drew lines along temple orientations and then used the GRASS azimuth functionality (see https://grass.osgeo.org/grass82/manuals/v.to.db.html). The third predictor was area in square metres estimated with GIS software on the basis of mapped polygons corresponding to visible temple footprints in remotely sensed images. The fourth and fifth predictors were presence/absence variables pertaining to whether or not a given temple had a spatially associated principal reservoir and a moat, respectively. Finally, the remaining sixth through eleventh predictors were presence/absence variables indicating whether a given material type had been identified at a given temple during ground survey work. The possible material types were ”sandstone”, ”pink sandstone”, ”laterite”, ”brick”, ”*thma phom*”, and ”other”, and any given temple may have had none, any, or all types present.

### Hybrid GLM-GSSL approach

As mentioned, Klassen et al.^11^ merged GLM-based predictions of temple foundation dates with the predictions derived from GSSL label propagation. The GLM portion of the hybrid model was a straightforward linear regression. For the present study, we used a simple Gaussian regression model to predict temple dates. The model included the temple morphology variable as a factor with an arbitrary category left out to form the baseline—effectively, the model intercept. The temple area variable was logged, and any temples with missing variables were dropped prior to estimating the model’s parameters. Importantly, the GLM approach requires that each undated temple has no missing predictor variables. Therefore, the GLM model was first trained on the set of complete cases (i.e. dated temples with no missing predictor variables) and then used to predict dates for undated temples that also had no missing predictors.

Producing predictions with GSSL label propagation required further consideration. As mentioned, label propagation is a technique that uses structure in a graph-based representation of a dataset containing labelled and unlabelled observations to predict labels for the unlabelled data. A graph is simply a network composed of nodes connected by edges (lines between the nodes)^27^. Each temple in the graph representation would be a node in the network. For our purposes, each node is then connected to every other node by an edge, and the edges are given weights proportional to the similarity between the temples. The similarity metric defines the structure of the graph and ultimately determines how labels (dates in our case) are propagated over the graph from labelled to unlabelled nodes. The similarity metric is, therefore, the foundation of the GSSL label propagation method.

Similarity refers to the distance between observations in a multidimensional space where each dimension is defined by one of the predictor variables, and it is defined slightly differently for the categorical and continuous predictors^11^. For the continuous variables (i.e. azimuth and area), the distance is a squared difference. As a result, the similarity, $s_{i,j}$, for a continuous variable *x*, between the $i{th}$ and $j{th}$ temples is defined as follows,

1 $$\begin{aligned} s_{i, j}&= 1 - (f(x_j) + f(x_i))^2 , \end{aligned}$$

2 $$\begin{aligned} f(x)&= \frac{x}{max\{x_1, x_2, ..., x_n\}} , \end{aligned}$$

where the function, $f(\cdot )$, scales the given variable to between 0 and 1, and *n* refers to the number of observations. As the equations indicate, the lowest similarity between any pair of temples on one single continuous dimension would be 0 (e.g. $f(x_i) = 0$ and $f(x_j) = 1$ or vice versa), and the highest similarity would be 1 (i.e. $f(x_i) = f(x_j)$). Missing continuous variables were assigned a value of 0.5, corresponding to half the relevant variable’s maximum value.

For the categorical predictors, similarity is defined by logical equality. Therefore, the similarity, $s_{i,j}$, for a categorical variable, *y*, between the $i{th}$ and $j{th}$ temples is defined as follows,

3 $$\begin{aligned} s_{i, j}&= {\left\{ \begin{array}{ll} 0, &{} \text {if}\ y_i \ne y_j \\ 1, &{} \text {otherwise} \end{array}\right. } \end{aligned}$$

Missing categorical variables were treated as an additional, separate category. In such cases, missingness (i.e. $\text {NA}$) would be considered equal to missingness and different from non-missingness—thus, $\text {NA} = \text {NA}$; $\text {NA} \ne 0$; and $\text {NA} \ne 1$.

These two types of similarity values were then added together to produce a total similarity between a given pair of temples. The minimum total similarity between any pair of temples could be 0 and the maximum could be 11 corresponding to the total number of predictor variables being compared.

To represent the graph mathematically, label propagation algorithms make use of a “graph Laplacian”^27^. A graph Laplacian is a square matrix where the diagonal elements indicate the degree of connectedness between a given node and all the others in the graph, and the off-diagonal elements indicate whether a given pair of nodes is connected. For label propagation, the matrix is modified such that its elements refer to similarities instead of connectedness. Following Klassen et al.^11^, the diagonal elements in our graph Laplacian indicate the overall similarity between a given unlabelled temple and all other labelled and unlabelled temples in the dataset. The off-diagonal elements contained the pairwise similarities between a given pair of undated temples.

The graph Laplacian was then entered into a system of linear equations as follows,

4 $$\begin{aligned} \begin{bmatrix} s_{1} &{} -u_{1,2} &{} \dots &{} -u_{1,n} \\ -u_{2,1} &{} s_{2} &{} \dots &{} -u_{2,n} \\ \vdots &{} \vdots &{} \ddots &{} \vdots \\ -u_{n,1} &{} -u_{n,2} &{} \dots &{} s_{n} \end{bmatrix} \begin{bmatrix} \tau _{1} \\ \tau _{2} \\ \vdots \\ \tau _{n} \end{bmatrix} = \begin{bmatrix} l_{1,1} &{} l_{1,2} &{} \dots &{} l_{1,m} \\ l_{2,1} &{} l_{2,2} &{} \dots &{} l_{2,m} \\ \vdots &{} \vdots &{} \ddots &{} \vdots \\ l_{n,1} &{} l_{n,2} &{} \dots &{} l_{n,m} \end{bmatrix} \begin{bmatrix} d_{1} \\ d_{2} \\ \vdots \\ d_{m} \end{bmatrix} \end{aligned}$$

where the first *n* by *n* matrix is the graph Laplacian itself—*n* referring to the number of undated temples. As mentioned, its diagonal elements, $s_i$, refer to the total similarity between the *i* *th* undated temple ($i \in {1, 2, \dots , n}$) and all other temples (dated or not). The off-diagonal elements, $u_{i,j}$, refer to the similarity between the $i{th}$ undated temple and $j{th}$ undated temple ($i, j \in {1, 2, \dots , n} | i \ne j$). The subsequent column vector in the equation contains *n* $\tau$-elements, each referring to the estimated foundation date of an undated temple. Next is an *n* by *m* matrix whose elements each refer to the similarity between the $i{th} | i \in {1, 2, \dots , n}$ undated temple and the $j{th} | j\in {1, 2, \dots , m}$ dated temple, where *m* refers to the number of dated temples. Finally, the last column vector contains *m* elements, $d_{i}$, that refer to the date of the $i{th} | i \in {1, 2, \dots , m}$ dated temple. The dot product of the matrix and vector on the right-hand side of the equation results in another length-*m* column vector whose elements would be a weighted sum of known temple dates—weighted, that is, by the similarity between a given undated temple and each dated one (the elements of the preceding matrix rows). Solving this system for $\tau$ produces the GSSL label propagation temple date estimates. They are the best-fitting date estimates in a least-squares sense. Intuitively, the equations indicate that each estimate represents a weighted average where the weighting is done according to similarities among the dated and undated temples.

Following Klassen et al.^11^, we then combined the GLM and GSSL-label propagation estimates. Klassen et al.^11^ found that GLM yields a lower mean absolute deviation than GSSL-label propagation. It was, therefore, reasoned that GLM-based estimates should be used where possible and GSSL estimates otherwise. So, for the present study, GLM estimates were produced for temples that had the full suite of predictor variables available, as described earlier, and then the entire dataset was used to produce estimates with the GSSL approach. Then, the GSSL estimates for temples with no missing variables were replaced with their corresponding GLM-based estimates.

### Bayesian approach

The alternative Bayesian approach we explored is a straightforward regression model. Like any regression, our dependent variable—temple foundation date—is reasoned to be a function of our predictors, e.g. temple area, azimuth, presence/absence of a moat. The model can be thought of as explaining variation in temple foundation date across a given sample of temples and, so, it can be used to “predict” the foundation date of an undated temple given the same predictors. A significant advantage of a Bayesian regression model is that it can easily accommodate missing values and, crucially, uncertainty about the true values of those missing variables. This is possible because Bayesian models are defined probabilistically and make use of prior information, which means we can quantify the uncertainty we have about missing values by reasoning about their distribution, given our domain knowledge^29^. Other relevant uncertainties, like measurement errors and uncertainty about the relationship between predictors and the dependent variable(s), can also be explicitly and quantitatively included in the model. These uncertainties are then propagated into the posterior distributions for the model’s estimated parameters and predictions.

The model we used represents the temple foundation date as a normally-distributed random variable, the mean of which is a linear combination of scaled predictor variables. $\tau _i$ refers to the foundation date of the $i{th}$ temple, where $i \in \{1, 2, \dots , n\}$ and *n* refers to the number of temples. We can refer to the mean date as $\mu _i$ and the standard deviation as $\sigma$. Then, more formally, each temple date can be represented as follows,

5 $$\begin{aligned} \tau _i&\sim \mathscr {N}({}_{\tau }\mu _i,{}_{\tau }\sigma ) , \end{aligned}$$

6 $$\begin{aligned} {}_{\tau }\mu _i&= \textbf{X}_i\varvec{\beta } , \end{aligned}$$

7 $$\begin{aligned} \beta _j&\sim \mathscr {N}({}_{\beta }\mu _j, {}_{\beta }\sigma _j), \end{aligned}$$

8 $$\begin{aligned} {}_{\tau }\sigma&\sim \mathscr {U}(l, u) , \end{aligned}$$

where $\mathscr {N}(\cdot )$ refers to the Normal distribution. Note that $\mu$ and $\sigma$ in Eqs. (5) and (7) refer to parameters for different variables and they are not the same, as indicated by the pre-subscripts. Then, $\textbf{X}\varvec{\beta }$ is the linear model containing the predictor variables as columns in the matrix $\textbf{X}$ and a column vector, $\varvec{\beta }$, of regression coefficients. Each *j* element of the $\varvec{\beta }$ corresponds to a given predictor variable and $\textbf{X}_i$ refers to the $i{th}$ row of $\textbf{X}$. The parameter ${}_{\tau }\sigma$ represents the standard deviation of the distribution of probable temple foundation dates, $\tau$, which reflects the uncertainty about the relationship between the predictors and temple date. As Eq. (6) indicates, the mean of the temple date distribution for a given temple is a linear combination of predictor variables for the $i^{th}$ temple weighted by the regression coefficients. As with most regression models, ${}_{\tau }\sigma$ and $\varvec{\beta }$ parameters are estimated from the data and given priors (Eqs. 7 and 8). For the latter, a uniform prior was used and denoted $\mathscr {U}(l, u)$, where *l* is a lower bound and *u* an upper bound.

Defining the mean, $\mu$, of this normally-distributed random variable took careful consideration because, as with the GSSL model, we needed to treat the predictors differently depending on their type. We also needed to come up with suitable prior distributions for each and then combine the predictors into a single linear combination that defines $\mu$. The priors were important in our model because they are the distributions that allowed us to impute missing values and thus define the probability that a given unobserved variable had a given true value. Here we will describe, in turn, each type of variable and the corresponding prior distribution we used.

Again, one of the predictors was temple morphology, a categorical variable. Every temple was assigned one of eight possible morphotypes based on a visual analysis of remote sensing data^11^. Since a given temple must have one and only one morphotype, this predictor was included in the Bayesian model as an index variable, which means that the model is eight separate regression models estimated simultaneously. This approach is commonly used to model categorical variables, and there are certain advantages to going this route instead of treating morphotype as, for instance, a set of dummy variables and then either dropping the standard regression intercept or one of the morphotype dummies from the analysis (to avoid perfect multicolinearity) [Ref.^29^, 155]. The results, however, would be mathematically equivalent either way.

An index variable is one where the index (subscript) used to identify a variable in the model is itself variable [Ref.^29^, 155]. For present purposes, that means that the term in the model referring to the impact of morphotype on temple date has eight possible indices. Morphotype is then represented by $\psi _m$ where *m* is the index variable. Then, we chose a uniform categorical prior for *m* defined by a Dirichlet distribution. The Dirichlet distribution is a multivariate generalization of the Beta distribution [Ref.^28^, 585]. Its realizations (outcomes) are vectors of values, usually denoted $\textbf{k}$, each element (*k*) of which has to be between 0 and 1, and all of which must sum to 1. The Dirichlet is parameterized by another vector of values, often denoted $\varvec{\alpha }$. The Dirichlet distribution is a natural choice as a prior for a categorical distribution where one and only one category can be chosen at a time because the probability that a given category is obtained is represented by the corresponding element of $\textbf{k}$—think a weighted m-sided die where the weights are given by $\textbf{k}$. The hyperparameter, $\varvec{\alpha }$, can be estimated from the data or defined explicitly on the basis of prior knowledge. The outcome vector, $\textbf{k}$, in our case, corresponded to the frequency of morphotypes, which we estimated from the data alongside other parameters during MCMC. Mathematically, the morphotype variable and its prior were defined as follows,

9 $$\begin{aligned} \psi _m&\sim \mathscr {N}({}_{\psi }\mu , {}_{\psi }\sigma ) \end{aligned}$$

10 $$\begin{aligned} m&\sim \mathscr {C}(\textbf{k}) \end{aligned}$$

11 $$\begin{aligned} \textbf{k}&\sim \mathscr {D}(\varvec{\alpha }) \end{aligned}$$

where $\mathscr {C}(\textbf{k})$ is the categorical distribution, the vector $\textbf{k}$ contains the probabilities of each category (morphotype) of which there were eight, $\mathscr {D}(\varvec{\alpha })$ is the Dirichlet distribution, and $\varvec{\alpha }$ is its parameter vector. Modelling the morphotype predictor in this way means that we can modify Eq. (6) slightly as follows,

12 $$\begin{aligned} \mu _i&= \psi _m + \textbf{X}_i\varvec{\beta } . \end{aligned}$$

In this equation, $\psi _m$ acts like an intercept and is free to vary among temple morphotypes. Importantly, the morphotype variable would now be excluded from the matrix of predictors because the effect of morphotype on date predictions is represented by the $\psi _m$ term. In the event that the morphotype is missing for a given temple, it can be imputed by drawing probable values for *m*, the index variable, from the distribution defined by Eq. (10).

The second type of predictor in the model was continuous, comprising the azimuth and area variables. These two variables entered into the regression as columns in the $\textbf{X}$ matrix from Eq. (12). In order to impute missing values, we applied a separate prior for each one. For the azimuth prior, we used a uniform distribution bounded by 1 on the low end and by 360 on the upper end. This reflects the fact that azimuth—degrees clockwise from North—is a bounded variable. Of course, it does not account for the fact that $0 = 360$, or that the boundaries used exclude potential azimuths between 360 and 1 degree. However, none of the temples in the dataset with azimuth entries have orientations between 360 and 1 degree, with the vast majority tightly clustered around a south-east orientation. For area, we used a log-normal prior with a log-mean of 7.7 and log-standard-deviation of 1.02, values chosen based on an examination of the distribution of logged areas for the temple data. This prior reflects the fact that the distribution of temple areas is highly skewed and always positive, with a few massive temples pulling the tail of the distribution upwards. We can write these priors as follows,

13 $$\begin{aligned} \textbf{X}_{\text {azimuth}}&\sim \mathscr {U}(l = 1, u = 360) \end{aligned}$$

14 $$\begin{aligned} \log _{n}(\textbf{X}_{\text {area}})&\sim \mathscr {N}(\mu = 7.7, \sigma = 1.02) \end{aligned}$$

The rest of the predictors were binary. To reiterate, these were presence/absence variables indicating whether the following were present at a given temple: primary reservoir, moat, sandstone, pink sandstone, laterite, brick, *thma phom*, and other (building material). Unlike morphotype, a given temple may have had any, all, or none of these traits in any combination. There was, therefore, no concern about perfect collinearity. Thus, we opted to model these predictors as binary variables and used a Bernoulli distribution as the prior for each one. A random variable characterized by a Bernoulli distribution has two possible outcomes, 1 or 0 (coin flips). The distribution has one parameter, $\theta$, which is the probability of a “success”, or obtaining a 1. Rather than explicitly determining $\theta$ for each variable, we used a Beta distribution prior and estimated each $\theta$ from the data during MCMC. Nevertheless, the posterior distribution for a given $\theta$ would be approximately the empirical frequency of the relevant trait in the database. These presence/absence data and their priors were as follows (for the $j{th}$ variable),

15 $$\begin{aligned} \textbf{X}_j&\sim \mathscr {B}\text {ern}(\theta _j) \end{aligned}$$

16 $$\begin{aligned} \theta _j&\sim \mathscr {B}\text {eta}(a_j, b_j). \end{aligned}$$

### Model comparison

We tried to ensure a fair comparison between the two approaches. There are potentially many ways to make such a comparison, which could focus on explanatory power, predictive power, ease of use, and/or computational requirements. Going into the study, however, we already knew that the GLM-GSSL hybrid was oriented more toward prediction than explanation. As a result, our main point of comparison quantitatively and analytically was predictive power.

Following Klassen et al.^11^, we used mean absolute deviation (MAD) to compare the predictive performance of the two approaches. The MAD statistic was calculated during leave-one-out cross-validation analyses for each approach. Leave-one-out cross validation (LooCV) is a common method for estimating predictive power. It is performed by dropping an observation (a temple, in this case) from a training dataset, using the remaining data to estimate model parameters, and then making a prediction for the dropped observation [Ref.^43^, 241–245]. After a prediction is made, the difference (deviation) between the prediction and the true observed value for a given dropped data point is calculated. Then the dropped observation is returned to the training dataset, and the process is repeated for each observation in turn. This results in a list of differences, one for each observation in the training dataset. The mean of these differences is calculated, which produces an estimate for average out-of-sample predictive error of the relevant model. The mean of the deviations (the MAD) and the distribution of deviations for the two approaches were compared to see which, if either, approach produced better average out-of-sample predictions. For the Bayesian model, we used the mean of the posterior age estimates from the relevant MCMC sample chain for the given dropped temple to make the comparisons.

### Variable importance

As noted above, one of the key benefits of using a standard regression model, like the Bayesian approach, is that we can evaluate the relative importance of the predictor variables. To investigate the relative importance of variables in the Bayesian model, we used Reversible-Jump MCMC (RJ-MCMC)^44^. RJ-MCMC adds a layer of complexity to a typical Bayesian MCMC in order to explore the effect of including and excluding potentially important variables at random.

To run a RJ-MCMC, each predictor variable is multiplied by an indicator variable—a variable that takes on a value of 0 or 1^44^. The value is drawn randomly each MCMC iteration for each variable and included in the model’s total likelihood. It is like adding a switch to each variable that randomly determines whether a given variable is included in a given iteration. A value of 1 means that a given variable has whatever effect would normally be determined by the product of its value and relevant model parameter (e.g. predictor multiplied by regression coefficient), whereas an indicator value of 0 means the given variable has no impact on the model because it is multiplied by 0.

These indicator variables are tracked throughout the simulation along with the other model parameters and stored in their own MCMC chains. In the end, the frequency of 1’s in a given indicator variable’s chain indicates the ”inclusion probability” for the relevant variable. Variables with high inclusion probability are relatively more important for improving the model’s overall likelihood than those with low probabilities. They are also, therefore, the most important for improving the predictions of the model because they are contributing the most to the model’s overall fit. We used RJ-MCMC to estimate the parameters of the Bayesian model for a training dataset that included only temples with no missing data. We then extracted the posterior inclusion probabilities for each predictor and examined the values.

At the same time, we were able to directly examine the impact of temple morphology on the age estimates. As explained, the morphotypes entered into the Bayesian model as an index variable, which ultimately acted as the intercept in a set of parallel regressions. As a result, the posterior distribution for each morphotype parameter indicated the marginal mean effect of that morphotype on temple age. We plotted the posterior distributions associated with each morphotype in order to examine their individual contributions to temple age for each type—effectively, the average baseline foundation date associated with each morphotype.

### Visualizing chronological uncertainty

Finally, we used the Bayesian model’s posterior estimates of temple foundation dates to visualize the historical trajectory of temple foundation events through time and space. For the temporal dimension, we counted the number of temples founded in each of the 7 century-long periods from 700–1400 CE. The chronological uncertainty in estimated foundation dates meant that there would be a distribution of possible counts for each period. The temple foundation date estimates (posteriors from the Bayesian model) in the MCMC chains were used to estimate these distributions. After a burn-in period was discarded, we used the set of sampled dates produced by the MCMC in a given iteration as one probable set of foundation dates. Those dates were binned into the century-long period, and the number of dates falling into each bin were then counted to produce a single probable count sequence of temple foundation events. This process was repeated for each MCMC iteration sample, producing tens of thousands of probable sequences. We produced a box-and-whisker plot for each temporal bin, which represented the distribution in each bin.

We then used an experimental approach to visualizing the temporally uncertain spatial process of temple foundation events. As explained, the posterior distributions from the Bayesian model represented the uncertainty around the predicted dates, which were by definition Gaussian (see Eq. (5)). So, we used the means and standard deviations of the MCMC samples for a given temple to estimate normal density functions for each predicted date. These functions were then used to define an opacity parameter for symbols used to represent the temples in an interactive online map application. We used the Shiny^45^ R framework for the application and an R package for mapping spatial data called leaflet^46^. Using a slider in the app interface, the user can select a date, which is then used to calculate the opacity from each predicted temple date’s density function. That way, sliding the slider changes the opacity of the temples’ symbols plotted on the map. Higher opacity values indicate higher posterior densities for the predicted dates—i.e. more probable foundation dates—at the time corresponding to the slider position. At the same time, temples were colour coded according to the position of their posterior date mean relative to the temporal position of the slider. If a given temple’s estimated mean foundation date was earlier than the date indicated by the slider, the corresponding symbol would be blue, and if it were later the symbol would be red. Thus, transparency indicated temporal uncertainty, and colour indicated the direction of the most probable foundation date on the timeline relative to the slider position. Interested readers can run the R script in the archived repository (https://zenodo.org/badge/latestdoi/560830491) to produce the app and use it interactively. To represent this information statically, we also produced a sequence of snapshots for comparison.

### Software

All analyses were conducted in R^47^. For the GLM model we used the core R function, “R::glm”. We then used the generic “R::solve” function in a script to solve the linear system detailed above for the GSSL-label propagation model. We used the R package Nimble^48,49^ to write the Bayesian model and run an MCMC simulation to estimate model parameters. The simulation was run multiple times to ensure consistency, involving at least 50,000 iterations each time. We then used a standard convergence diagnostic—the Geweke diagnostic^50^—to identify potential non-stationarity in MCMC chains and look for convergence problems.

Several other packages were important for analysis and plotting. These included coda^51^, tidyverse^52^, ggplot2^53^, ggpubr^54^, GGally^55^, and readxl^56^, and progress^57^. We also used the interactive app development tool, shiny^45^, along with spatial analysis packages sp^58^ and maps^59^ to plot temple locations and create a browser-based map app for exploring temple foundation dates with chronological uncertainty. The data and code necessary for replication and evaluation are available on Github at https://github.com/wccarleton/angkortemples and the repository will be archived with Zenodo (https://zenodo.org/badge/latestdoi/560830491).

## Data Availability

The datasets generated during and/or analysed during the current study are available in the Zenodo repository, https://zenodo.org/badge/latestdoi/560830491.
